# Effectiveness of Interventions to Increase Colorectal Cancer Screening Among American Indians and Alaska Natives

**DOI:** 10.5888/pcd17.200049

**Published:** 2020-07-16

**Authors:** Donald Haverkamp, Kevin English, Jasmine Jacobs-Wingo, Amanda Tjemsland, David Espey

**Affiliations:** 1 Centers for Disease Control and Prevention, Division of Cancer Prevention and Control, Albuquerque, New Mexico; 2 Albuquerque Area Indian Health Board, Albuquerque, New Mexico

## Abstract

**Introduction:**

Screening rates for colorectal cancer are low in many American Indian and Alaska Native (AI/AN) communities. Direct mailing of a fecal immunochemical test (FIT) kit can address patient and structural barriers to screening. Our objective was to determine if such an evidence-based intervention could increase colorectal cancer screening among AI/AN populations.

**Methods:**

We recruited study participants from 3 tribally operated health care facilities and randomly assigned them to 1 of 3 study groups: 1) usual care, 2) mailing of FIT kits, and 3) mailing of FIT kits plus follow-up outreach by telephone and/or home visit from an American Indian Community Health Representative (CHR).

**Results:**

Among participants who received usual care, 6.4% returned completed FIT kits. Among participants who were mailed FIT kits without outreach, 16.9% returned the kits — a significant increase over usual care (*P* < .01). Among participants who received mailed FIT kits plus CHR outreach, 18.8% returned kits, which was also a significant increase over usual care (*P* < .01) but not a significant increase compared with the mailed FIT kit–only group (*P* = .44). Of 165 participants who returned FIT kits during the study, 39 (23.6%) had a positive result and were referred for colonoscopy of which 23 (59.0%) completed the colonoscopy. Twelve participants who completed a colonoscopy had polyps, and 1 was diagnosed with colorectal cancer.

**Conclusion:**

Direct mailing of FIT kits to eligible community members may be a useful, population-based strategy to increase colorectal cancer screening among AI/AN people.

**Summary**

**What is already known about this topic?**

Reducing client and structural barriers can result in greater participation in colorectal cancer screening, when stool-based tests are used.

**What is added by this report?**

Direct mailing of fecal immunochemical test kits was an effective strategy to increase colorectal cancer screening participation at rural, tribally operated health care facilities.

**What are the implications for public health practice?**

Stool-based testing is often the most accessible colorectal cancer screening option at rural, tribally run health care facilities. Direct mailing of fecal immunochemical tests may increase colorectal screening at health care facilities that serve American Indian and Alaska Native populations.

**MEDSCAPE CME**

Medscape, LLC is pleased to provide online continuing medical education (CME) for this journal article, allowing clinicians the opportunity to earn CME credit.

In support of improving patient care, this activity has been planned and implemented by Medscape, LLC and *Preventing Chronic Disease*. Medscape, LLC is jointly accredited by the Accreditation Council for Continuing Medical Education (ACCME), the Accreditation Council for Pharmacy Education (ACPE), and the American Nurses Credentialing Center (ANCC), to provide continuing education for the healthcare team.

Medscape, LLC designates this Journal-based CME activity for a maximum of 1.00 *AMA PRA Category 1 Credit(s)™*. Physicians should claim only the credit commensurate with the extent of their participation in the activity.

All other clinicians completing this activity will be issued a certificate of participation. To participate in this journal CME activity: (1) review the learning objectives and author disclosures; (2) study the education content; (3) take the post-test with a 75% minimum passing score and complete the evaluation at http://www.medscape.org/journal/pcd; (4) view/print certificate.

**Release date: July 16, 2020; Expiration date: July 16, 2021**

**Learning Objectives**

Upon completion of this activity, participants will be able to:

Distinguish the overall rate of FIT kit return in the current study

Analyze variables associated with higher rates of FIT kit return

Compare mail-only and mail-only plus CHR strategies with usual care alone

Assess the effects of CHR on FIT kit return in the current study

EDITOR

Rosemarie Perrin

Editor

*Preventing Chronic Disease*

Disclosure: Rosemarie Perrin has disclosed no relevant financial relationships.

CME AUTHOR

Charles P. Vega, MD

Health Sciences Clinical Professor of Family Medicine

University of California, Irvine, School of Medicine

Irvine, California

Disclosure: Charles P. Vega, MD, has disclosed the following relevant financial relationships:

Served as an advisor or consultant for: Johnson & Johnson Pharmaceutical Research & Development, LLC; GlaxoSmithKline

Served as a speaker or a member of a speakers bureau for: Genentech, GlaxoSmithKline

AUTHORS

Donald Haverkamp, MPH

Centers for Disease Control and Prevention Division of Cancer Prevention and Control

Albuquerque, New Mexico

Disclosure: Donald Haverkamp, MPH, has disclosed no relevant financial relationships.

Kevin English, DrPH

Albuquerque Area Indian Health Board

Albuquerque, New Mexico

Disclosure: Kevin English, DrPH, has disclosed no relevant financial relationships.

Jasmine Jacobs-Wingo, MPH

Centers for Disease Control and Prevention

Division of Cancer Prevention and Control

Albuquerque, New Mexico

Disclosure: Jasmine Jacobs-Wingo, MPH, has disclosed no relevant financial relationships.

Amanda Tjemsland, BA

Centers for Disease Control and Prevention

Division of Cancer Prevention and Control

Albuquerque, New Mexico

Disclosure: Amanda Tjemsland, BA, has disclosed no relevant financial relationships.

David Espey, MD

Centers for Disease Control and Prevention

Division of Cancer Prevention and Control

Albuquerque, New Mexico

Disclosure: David Espey, MD, has disclosed no relevant financial relationships.

## Introduction

Colorectal cancer (CRC) is the second leading cause of death from cancer among American Indian and Alaska Native (AI/AN) men and third among AI/AN women (1). Although screening has been shown to reduce death rates, the percentage of people up to date with CRC screening is low in many AI/AN communities. Less than half (48.4%) of AI/AN adults aged 50 to 75 were up to date with CRC screening in 2015 (2).

The US Preventive Services Task Force (USPSTF) recommends stool-based tests and direct visualization tests (colonoscopy, flexible sigmoidoscopy, or virtual colonoscopy) for CRC screening. (3). In health care systems with limited capacity to provide direct-visualization screening tests, stool-based tests such as high-sensitivity, guaiac-based fecal occult blood tests (FOBT) and fecal immunochemical tests (FIT) are often the most accessible options for CRC screening. However, various patient and structural barriers exist to completing FOBT and FIT: geographic isolation, lack of a regular health care provider, failure of providers to recommend screening, lack of clinical tracking and reminder systems, lack of transportation, embarrassment, privacy concerns, distrust of the health care system, and insufficient knowledge about CRC, its risk factors, and screening recommendations (4). Many of these barriers can be mitigated. According to the Community Preventive Services Task Force, there is sufficient evidence that using patient reminders and small media (eg, letters, pamphlets, brochures, flyers) can increase CRC screening with stool tests (5). Reducing structural barriers (eg, eliminating or simplifying administrative procedures required for CRC screening, reducing time or distance for screening services) is also an effective way to increase the use of stool tests (6). Direct mailing of FOBT or FIT is an approach that can address both patient and structural barriers. Mailing FOBT or FIT kits to patients and providing outreach through telephone calls and home visits can reduce patient and structural barriers, and both have been shown to be effective strategies to improve participation in CRC screening in various underserved populations (7–10). The objective of our study was to determine if such evidence-based interventions could also lead to increased CRC screening among rural AI/AN populations.

## Methods

### Participant recruitment

We recruited 3 tribally operated health care facilities with which we had a previous working relationship to participate in our study. The selected facilities were in different tribal communities. At each facility, the clinic director used Resource and Patient Management System Query Manager (11) to generate a list of active clinic users (people who had obtained services at least once in the past 3 years), were aged 50 to 75, were not up to date with CRC screening per USPSTF criteria at the time the study began (had not had an FOBT or FIT in the past year, flexible sigmoidoscopy in the past 5 years combined with FOBT or FIT in the past three years, or colonoscopy in the past 10 years) (12), and had no history of CRC or total colectomy. These criteria were met by 1,288 people. Our study was approved by institutional review boards of the Centers for Disease Control and Prevention (CDC) and the 3 participating tribal health care facilities.

**Figure 1:**
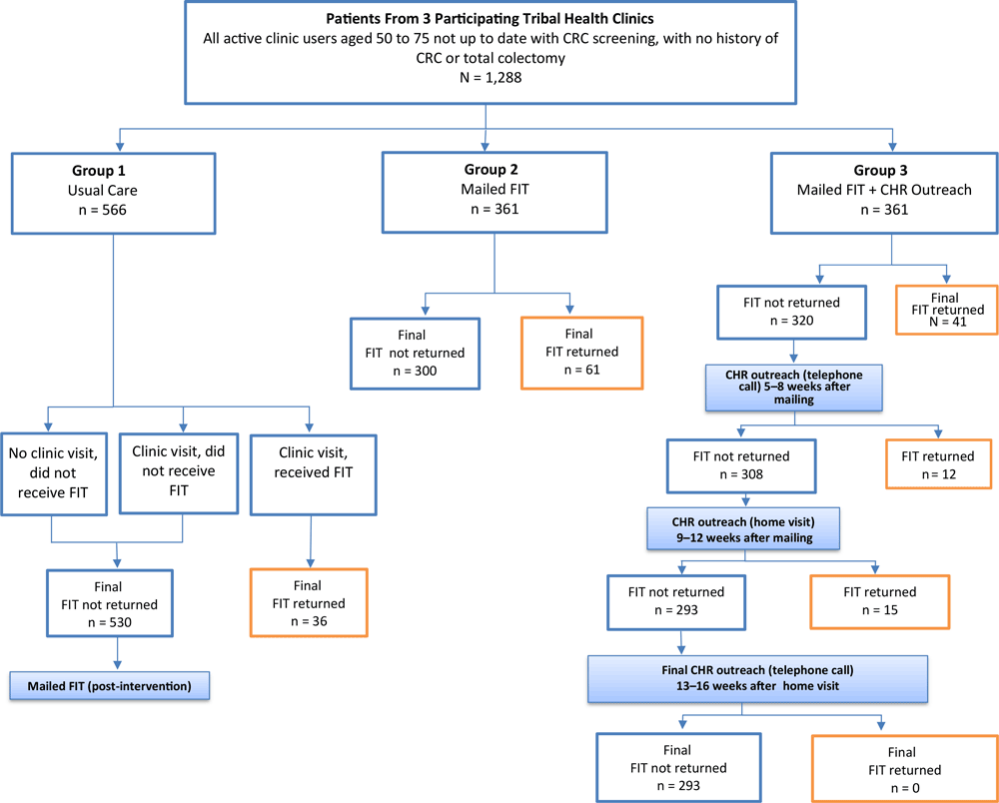
Participant selection, randomization, and outcomes in 3 study groups, intervention to increase colorectal cancer screening among American Indian and Alaska Native people (N = 1,288) served by 3 tribally operated health care clinics, April to November, 2014. Group 1, usual care, consisted of people who either did not visit the clinic, visited the clinic and did not receive a fecal immunochemical test (FIT) kit, or visited and received a FIT kit and instructions to complete at home. Group 2 participants received a FIT kit and completion instructions by mail. Group 3 participants received a mailed FIT kit and instructions, and nonrespondents received follow-up from a tribal community health representative after 4 weeks (by telephone), after 8 weeks (by home visit), and after 12 weeks by telephone. Abbreviations: CHR, community health representative; FIT, fecal immunochemical test.

**Study design.** At each facility, study participants were randomly assigned to 1 of 3 study groups: group 1 (the control or usual care group), in which participants visited the clinic with the same frequency as they would outside study conditions and received a FIT kit only if a provider recommended one; group 2 (mailing alone), in which participants were mailed FIT kits (Polymedco OC-Light), completion instructions (in English), a letter (in English) notifying them that they were due for CRC screening, and a prestamped, pre-addressed envelope for returning their completed FIT kit; and group 3 (mailing plus outreach), in which participants were mailed the same materials as group 2 and also received telephone and/or home visit follow-up from an American Indian Community Health Representative (CHR) if they did not return the completed test (Figure 1). At 2 study facilities, we randomized 133 CRC screening-eligible participants to each of the 3 groups, and included the remaining 205 screening-eligible patients at these 2 facilities in the usual-care group (124 people at one clinic and 81 people at the other clinic) (Table). Because 1 study clinic had a smaller patient population, we randomized all 285 CRC screening-eligible people at that facility equally among the 3 study groups (95 in each group). Providers were blinded to their patients’ involvement in the study or study group. We hypothesized that the percentage of eligible persons completing FIT in each of the 2 intervention groups would be significantly higher than the percentage completing FIT in the usual care group (group 1), and that the percentage completing FIT in the mail-out plus outreach group (group 3) would be significantly higher than the percentage completing FIT in the mail-out alone group (group 2).

**Table:** Colorectal Cancer Screening Interventions in 3 Tribally Operated Health Care Centers Using the Fecal Immunochemical Test (FIT), 3 Intervention Groups, April–November 2014^a^

Variable	Group 1, Usual Care^b ^(n = 566)	Group 2, Mailing Alone^c ^(n = 361)	Group 3, Mailing + Outreach^d ^(n = 361)	Total (N = 1,288)
**Center**				
1	257 (45.4)	133 (36.8)	133 (36.8)	523 (40.6)
2	95 (16.8)	95 (26.3)	95 (26.3)	285 (22.1)
3	214 (37.8)	133 (36.8)	133 (36.8)	480 (37.3)
**Age, y^e^**				
Mean, (standard deviation)	60.6 (7.0)	60.8 (6.8)	59.8 (6.7)	60.4 (6.9)
50–59	284 (50.3)	170 (47.2)	194 (53.7)	648 (50.4)
60–69	204 (36.1)	149 (41.4)	131 (36.3)	484 (37.6)
70–75	77 (13.6)	41 (11.4)	36 (10.0)	154 (12.0)
**Women**	291 (51.4)	179 (49.6)	200 (55.4)	670 (52.0)

a Values are number (percentage) unless otherwise indicated.

b No outreach apart from provider screening advice given during clinic visits.

c Mailing FIT kit with instructions for use.

d Mailing FIT kit with instructions for use. If no response, follow-up telephone call after 4 weeks, follow-up home visit after 8 weeks, and telephone call after 12 weeks.

e Values for 3 groups may not equal totals because some participants did not provide age.

**Intervention design.** We educated CHRs from each facility about CRC screening recommendations and our intervention protocol. We also informed clinic administrators and staff at each facility about the study. In April 2014, we mailed FIT kits, instructions for completion, an official letter from the clinic, and prestamped envelopes to participants in the intervention groups (groups 2 and 3). In August 2014, we mailed a follow-up letter to nonrespondents in groups 2 and 3 who had not yet returned kits, encouraging them to do so. The intervention period for all study groups was April 2014 through November 2014.

The outreach intervention protocol (group 3) instructed CHRs to make up to 5 attempts to contact by telephone all participants who had not returned their FIT kits within 4 weeks of the mailing; CHRs were to make up to 3 attempts to conduct a home visit to those who had not returned their FIT within 8 weeks, and up to 5 attempts to contact nonrespondents by telephone who had not returned the kits by the end of week 12 (Figure 1). If at first attempt a participant’s telephone number was found to be disconnected or incorrect, CHRs were to visit that participant’s home as the initial outreach activity. As part of their outreach, CHRs were to confirm that the participant received the mailed FIT kit (and provide another FIT kit if the participant did not receive the first), discuss the importance of CRC screening, review procedures for completing the FIT kit, encourage the participant to complete the FIT kit, answer questions, and offer to transport the completed FIT kit to the clinic laboratory.

**Data tracking procedures.** We created 2 databases to track results: 1 for laboratory staff to collect patient contact information and demographics, how and when FIT kits were disseminated and returned, and test results and another for CHRs to gather patient contact information and demographics, outreach type (telephone call or home visit), and other outreach details. Only clinic directors (or their designees), laboratory directors, and CHRs had access to the databases.

On-site clinic laboratories processed all completed FIT kits. Laboratory staff recorded FIT results in the participant tracking database and patient medical charts. Per standard operating procedures (3), clinic providers were instructed to refer any participant with a positive FIT result for colonoscopy.

**Data analysis.** Both the laboratory and CHR tracking databases were de-identified after the study intervention period, and the data files from all 3 facilities were merged. We used SPSS 22 (IBM Corp) software to perform Pearson χ^2^ testing to determine significant differences (*P* < .05) in FIT completion between study groups.

## Results

The mean age of the 1,288 study participants was 60, half were aged 50 to 59, and 52% were women. (Table). Overall, 12.8% (165/1,288) returned a completed FIT kit to their clinic, and FIT completion did not differ by sex (*P* = .52). The proportion who returned FIT kits increased with age: 10.8% (70/648) aged 50 to 59, 13.6% (66/484) aged 60 to 69, and 18.8% (29/154) aged 70 to 75 (*P* = .02). Most who completed FIT kits hand delivered them to the clinic (83.0%), whereas 16.4% used the pre-stamped, pre-addressed envelope to return the kit by mail. Only one completed FIT kit (0.6%) was delivered to the clinic by a CHR.

**Figure 2:**
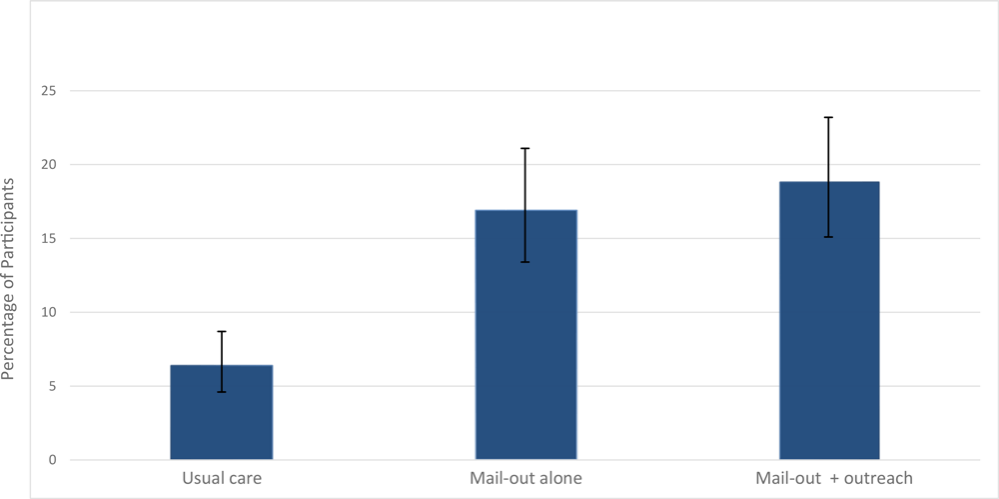
Percentage of participants who completed the fecal immunochemical test, by intervention group. Brackets indicate confidence intervals.

The percentage of returned FIT kits varied by study group (Figure 2). Among the participants who received usual care (group 1), 6.4% (36/566) completed their FIT kits at home and returned them to the clinic. In group 2 (mailing alone), 16.9% (61/361) returned the FIT kits, a significant increase over group 1 (*P* < .01). In group 3 (mailing plus outreach), 18.8% (68/361) returned FIT kits to the clinic, a significant increase over group 1 (*P* < .01), but not group 2 (*P* = .44) (Figure 2). Among those who returned a FIT kit, more women than men returned them in group 3 (50.0% vs 31.6%) and more men than women in group 2 (43.0% vs 31.4%) (*P* = .06).

**Table:** 

Intervention Group	% (95% CI)
Usual Care	6.4 (4.6–8.7)
Mail-out alone	16.9 (13.4–21.1)
Mail-out plus outreach	18.8 (15.1–23.2)

Among all group 3 participants, 11.4% (41/361) returned their FIT kit during the 4-week period before any CHR outreach began. After receiving a single round of CHR outreach, an additional 3.3% (12/361) returned their FIT kit. Following a second round of CHR outreach, another 4.2% (15/361) returned their FIT kit. No additional FIT kits were returned to the clinics among participants who received a third round of outreach (Figure 1). Because of delays in the implementation of the CHR intervention, varying rounds of outreach were still being conducted with participants in the months following the mailing of a reminder letter at the end of the intervention timeframe. Following the reminder letter mailing, 17 people in group 3 and 13 in group 2 returned their FIT kits. Of these 17 from group 3, 10 also received CHR outreach during that time. Overall, 51.5% (35/68) of group 3 participants who returned their FIT kits received outreach of some kind (telephone call and/or home visit) during the intervention period, including a few who received telephone call or home visit outreach even though they had already returned their FIT kit. Of the 293 participants in group 3 who did not return FIT kits, 76.8% (225/293) received outreach of some kind (telephone call and/or home visit) during the intervention period.

Of the 165 FIT kits returned, 39 had a positive result; all 39 were referred for colonoscopy, and 23 of the 39 completed the colonoscopy. Results of those colonoscopies showed that 12 participants had polyps, and 1 participant was diagnosed with CRC.

## Discussion

Our study showed that a significant increase in CRC screening participation is possible in AI/AN communities by mailing FIT kits and instructions to eligible community members and providing easy options for returning the kits to the clinic. The addition of telephone and home visit outreach following the FIT mailing also increased screening compared with the usual care group in our study, but not significantly beyond the level attained by only mailing FIT kits. Results similar to ours were reported by Coronado et al (8), with post-intervention CRC screening rates of 26% among Hispanic patients who received mailed FOBT only and 31% in the group that received mailed FOBT plus telephone call and home visit outreach; both results were significantly higher than the 2% screened in the usual care group, but not significantly different from one another. Another study demonstrated that the addition of telephone calls to encourage screening and to address barriers did not result in increased FIT completion compared with just mailing a FIT kit with printed educational materials (13). In contrast, Walsh et al (7) reported that self-reported FOBT screening rates among Latinos and Vietnamese patients at 1-year follow up increased by 7.8% in the usual care group, 15.1% in an FOBT mailing and brochure group, and 25.1% in a mailing, brochure, and telephone counseling group. The differences were significant between usual care and each intervention and between the 2 intervention groups.

One possible reason that our study’s CHR outreach failed to significantly boost the FIT return percentage compared with mailing alone was the lack of the CHR intervention among many group 3 participants. Of those in group 3 who did not return their FIT, nearly 1 in 4 did not receive any outreach. This most likely occurred because of staff turnover during the study period and competing CHR job duties that limited the time available to implement the outreach as specified in the study protocol. In some instances, CHRs could not reach participants because of incorrect phone numbers or addresses — a common barrier to conducting community outreach. In a similar study by Jean-Jacques et al (14), 23% of participants had incorrect or nonfunctional telephone numbers. Lasser et al (15) reported that of those eligible for patient navigation, 25% could not be contacted after 8 to 11 telephone call attempts. When a tribal facility or health system chooses to use CHRs to assist with cancer screening, CHRs need to have designated time to focus on this task. Patient navigators hired in 1 facility in Alaska specifically to assist with CRC screening efforts dramatically increased the number of CRC patients’ first-degree relatives who completed screening (16). Future studies could seek to determine how much outreach is appropriate before reaching saturation. In our study, no additional FIT kits were returned after the second round of outreach.

Even though our study showed a significant increase in return of FIT kits from participants who received mailed kits compared with usual care, the percentage of mailed kits that were returned in both intervention groups combined (17.9%) was still low. Many reasons have been identified for nonresponse to a direct mailing of stool test kits, including fear of results, cost of follow-up colonoscopy, not having received the mailed test, concerns about mailing fecal matter, and forgetfulness (17). Cultural barriers in AI/AN communities, such as medical mistrust, may also be a factor (4). Most participants in our study hand-delivered their completed FIT to the clinic instead of using the mailing envelope. Concern over mailing fecal material could be investigated further in this population. Additionally, some study participants may have had a language barrier. In our study, all written information with the FIT kit was in English. One alternative is to send out wordless instructions (eg, images/photographs) for completing the mailed FIT kit (18).

When stratified by age, our results showed that the percentage of returned FIT kits was highest at older ages. In the overall US population, CRC screening has been shown to be about 18% at age 50, increasing to 28% by age 51 (19). AI/AN people are less likely than other racial/ethnic groups to initiate screening at the recommended aged of 50 (20) and are more likely to be diagnosed with CRC at ages younger than 50, compared with non-Hispanic white people (21). Providers serving AI/AN populations need to ensure that their patients begin screening at the appropriate age and continue screening at the correct intervals, depending on their chosen method of screening and CRC risk level.

A large percentage of participants who returned FIT kits in our study (24%) had a positive FIT result. In a study by Hubbard et al (22), the risk of having a false-positive result from an FOBT was significantly greater among AI/AN than white patients. The greater risk of false-positive results among AI/AN populations could be a result of using FOBT for both symptomatic and asymptomatic patients. Any facility considering implementing population-based screening with FOBT or FIT in a population that has not been screened previously may need to prepare for a higher-than-expected proportion of positive test results and secure a facility that can perform the necessary follow-up colonoscopies.

Screening with FOBT or FIT reduces mortality from CRC only if patients with positive results undergo a follow-up colonoscopy. In our study, 41% of those with positive FIT results did not receive a follow-up colonoscopy. Several documented reasons for not completing colonoscopy are competing health concerns, failure to respond to follow-up outreach telephone calls and mailings, refusal, moving, and comorbidities that preclude safe colonoscopy (23). Others have suggested that noncompliance may be due to a combination of factors at the patient, provider, and health systems levels (24). Stock et al (25) showed that a notification sent directly to FOBT-positive screening patients increased colonoscopy uptake. A telephone call reminder, in addition to a mailed notification, may also improve the acceptance rate of colonoscopy in patients with a positive FIT (26).

Our study had several limitations. We conducted the study in 3 Southwest tribal communities, so results are not generalizable to all AI/AN populations. CHRs were unable to carry out all outreach as directed by the study protocol, compromising the comparison in FIT return between groups 2 and 3. Finally, we cannot conclude that group 3 participants who returned FIT kits after the outreach did so as a proximal result of outreach instead of the mailing itself.

The elimination of structural barriers through direct mailing of FIT kits to eligible community members is a useful, population-based approach to increase CRC screening among AI/AN people. The role of CHRs in improving CRC screening efforts could be studied further. Identifying interventions that increase the use of FOBT or FIT among AI/AN populations could have important implications for the uptake of CRC screening services and for decreased CRC mortality.

## Post-Test Information

To obtain credit, you should first read the journal article. After reading the article, you should be able to answer the following, related, multiple-choice questions. To complete the questions (with a minimum 75% passing score) and earn continuing medical education (CME) credit, please go to http://www.medscape.org/journal/pcd. Credit cannot be obtained for tests completed on paper, although you may use the worksheet below to keep a record of your answers.

You must be a registered user on http://www.medscape.org. If you are not registered on http://www.medscape.org, please click on the “Register” link on the right hand side of the website.

Only one answer is correct for each question. Once you successfully answer all post-test questions, you will be able to view and/or print your certificate. For questions regarding this activity, contact the accredited provider, CME@medscape.net. For technical assistance, contact CME@medscape.net. American Medical Association’s Physician’s Recognition Award (AMA PRA) credits are accepted in the US as evidence of participation in CME activities. For further information on this award, please go to https://www.ama-assn.org. The AMA has determined that physicians not licensed in the US who participate in this CME activity are eligible for *AMA PRA Category 1 Credits™*. Through agreements that the AMA has made with agencies in some countries, AMA PRA credit may be acceptable as evidence of participation in CME activities. If you are not licensed in the US, please complete the questions online, print the AMA PRA CME credit certificate, and present it to your national medical association for review.

## Post-Test Questions

### Study Title: Effectiveness of Interventions to Increase Colorectal Cancer Screening Among American Indians and Alaska Natives

#### CME Questions

1. What overall percentage of patients returned a completed FIT kit, according to results of the current study by Haverkamp and colleagues?
  - 13%
  - 27%
  - 41%
  - 55%
2. Which of the following variables had a positive and significant impact on the rate of returned FIT kits in the current study by Haverkamp and colleagues?
  - Female sex and younger age
  - Older age only
  - Female sex only
  - Neither sex nor age influenced the rate of returned FIT kits
3. Which of the following statements reflects one of the main outcomes of the current study?
  - There was no improvement in the mail-out alone or mail-out plus outreach cohorts vs the usual care cohort
  - Only the mail-out plus outreach cohort had a significant improvement in FIT kit return rates vs usual care
  - Both the mail-out alone and mail-out plus outreach cohorts had similar improvements in FIT kit return rates vs usual care
  - There was a significant and stepwise improvement in FIT kit return rates from usual care to mail-out alone to mail-out plus outreach
4. Which of the following statements regarding the use of CHRs in the current study is most accurate?
  - Most patients who returned FIT kits in the mail-out plus CHR cohorts did so after speaking with a CHR
  - 95% of patients in the mail-out plus CHR cohort communicated with a CHR
  - Only one FIT kit was returned by a CHR on behalf of a patient
  - Yields of return were good even on the third round of outreach from CHRs
